# Supplementary material for: Acceptance and compliance with micronutrient powder (MNP) among children aged 6–23 months in northern Nigeria
Source: PLOS Glob Public Health. 2022 Oct 17;2(10):e0000961. doi: 10.1371/journal.pgph.0000961 (PMC10022258; doi:10.1371/journal.pgph.0000961)
Supplement: S1 Dataset — (ZIP) [file pgph.0000961.s008.zip › S8_Dataset/Spot Checks.zip!/Spot Checks/090715_A_GA_ID_2_SC.pdf]

SPOT CHECK RECORDING FORM

State: __ADAMAWA___LGA: __GANYE__ Community: ____GURUM-NUVAN__

Sex of index child: __MALE__ Age of index child (in months): ____17MONTHS_____

Spot Check Information

1. **Date of MNP distribution to household** (circle option below based on LGA name above)**:**
    - **Kebbi:** BirninKebbi&Jega LGA: **June 16**
    - **Kebbi:** Danko-wasagu: **June 17**
    - **Adamawa:**Song, Ganye: **(June 18)**
    - **Adamawa:**Guyuk **June 19**
2. **Date of Spot Check Observation: __8th July, 20I5_____**
3. **Number of sachets remaining** (Ask the caregiver to show you and count the specific number of sachets remaining)**:**

    41

4. **Number of sachets that should remaining one MNP sachet per day** (Analyst only)**:**

    42

5. **Number of sachets remaining / number of sachets should be remaining**(Analyst only)**:**

$$\frac{41}{42} \times 100 = 97.6\ \%$$

Probes

Question 1. You have **XX** MNP sachets remaining. Explain how easy or difficult it was to use the MNP product on a daily basis for your child 6 – 23 months? (Circle one answer)

Very Difficult **Difficult** **(Easy)** **Very Easy**

Question 2. Describe any specific challenges that you faced that made the MNP difficult to use on a daily basis?The only challenge was that I don't usually know what to do with the left over kunu mixed with the mnp……whether to throw it away or keep it and give the child later on?

Question 3. Describe specific aspects of the MNP product that you or your child enjoyed that made it easier to use on a daily basis?

MNP is improving my child health because he is eaten food more than before.

Question 4. What questions do you have about the MNP based on your experience using it?

Is it only in kunu can this mnp be mixed?

Question 5. What specific recommendations do you have that we can do as a program to make the MNP easier to use on a daily basis?

MNP is a good because my child can now eat food not like before then he only suck breast and milk.
